# Implementation of Zebrafish Ontologies for Toxicology Screening

**DOI:** 10.3389/ftox.2022.817999

**Published:** 2022-03-11

**Authors:** Anne E. Thessen, Skylar Marvel, J. C. Achenbach, Stephan Fischer, Melissa A. Haendel, Kimberly Hayward, Nils Klüver, Sarah Könemann, Jessica Legradi, Pamela Lein, Connor Leong, J. Erik Mylroie, Stephanie Padilla, Dante Perone, Antonio Planchart, Rafael Miñana Prieto, Arantza Muriana, Celia Quevedo, David Reif, Kristen Ryan, Evelyn Stinckens, Lisa Truong, Lucia Vergauwen, Colette Vom Berg, Mitch Wilbanks, Bianca Yaghoobi, Jon Hamm

**Affiliations:** ^1^ Center for Health AI, University of Colorado Anschutz Medical Campus, Aurora, CO, United States; ^2^ Department of Biological Sciences, Bioinformatics Research Center, North Carolina State University, Raleigh, NC, United States; ^3^ Aquatic and Crop Resource Development Research Center, National Research Council of Canada, Halifax, NS, Canada; ^4^ aQuaTox-Solutions Ltd, Wallisellen, Switzerland; ^5^ Department of Environmental and Molecular Toxicology and the Sinnhuber Aquatic Research Laboratory, Oregon State University, Corvallis, OR, United States; ^6^ Department Bioanalytical Ecotoxicology, Helmholtz Centre for Environmental Research (UFZ), Leipzig, Germany; ^7^ Environmental Toxicology, Swiss Federal Institute of Aquatic Science and Technology (Eawag), Dübendorf, Switzerland; ^8^ Environment & Health, Vrije Universiteit Amsterdam, Amsterdam, Netherlands; ^9^ Department of Molecular Biosciences, University of California, Davis, Davis, CA, United States; ^10^ Environmental Laboratory, US Army Engineer Research and Development Center, Vicksburg, MS, United States; ^11^ Center for Computational Toxicology and Exposure, Biomolecular and Computational Toxicology Division, U.S. Environmental Protection Agency, Research Triangle Park, NC, United States; ^12^ Center for Human Health and the Environment, and Center for Environmental and Health Effects of PFAS, Biological Sciences, NC State University, Raleigh, NC, United States; ^13^ ZeClinics SL, Badalona, Spain; ^14^ Biobide USA, Cambridge, MA, United States; ^15^ Biobide, Donostia-San Sebastian, Spain; ^16^ Division of the National Toxicology Program, National Institute of Environmental Health Sciences, Durham, NC, United States; ^17^ Zebrafishlab, Department of Veterinary Sciences, University of Antwerp, Wilrijk, Belgium; ^18^ Integrated Laboratory Systems, LLC, Contractor supporting the National Toxicology Program Interagency Center for the Evaluation of Alternative Toxicological Methods, National Institute of Environmental Health Sciences, Durham, NC, United States

**Keywords:** phenotype, ontology, annotation, Danio rerio, zebrafish, endpoint

## Abstract

Toxicological evaluation of chemicals using early-life stage zebrafish (*Danio rerio*) involves the observation and recording of altered phenotypes. Substantial variability has been observed among researchers in phenotypes reported from similar studies, as well as a lack of consistent data annotation, indicating a need for both terminological and data harmonization. When examined from a data science perspective, many of these apparent differences can be parsed into the same or similar endpoints whose measurements differ only in time, methodology, or nomenclature. Ontological knowledge structures can be leveraged to integrate diverse data sets across terminologies, scales, and modalities. Building on this premise, the National Toxicology Program’s Systematic Evaluation of the Application of Zebrafish in Toxicology undertook a collaborative exercise to evaluate how the application of standardized phenotype terminology improved data consistency. To accomplish this, zebrafish researchers were asked to assess images of zebrafish larvae for morphological malformations in two surveys. In the first survey, researchers were asked to annotate observed malformations using their own terminology. In the second survey, researchers were asked to annotate the images from a list of terms and definitions from the Zebrafish Phenotype Ontology. Analysis of the results suggested that the use of ontology terms increased consistency and decreased ambiguity, but a larger study is needed to confirm. We conclude that utilizing a common data standard will not only reduce the heterogeneity of reported terms but increases agreement and repeatability between different laboratories. Thus, we advocate for the development of a zebrafish phenotype atlas to help laboratories create interoperable, computable data.

## Introduction

Zebrafish are a key animal model for toxicology because they are easily maintained and bred in the laboratory and they have rapid, easily observed development. Additionally, in the U.S., zebrafish embryos that have not yet begun to feed (<72 h post fertilization) are not subject to the Public Health Service Policy on Humane Care and Use of Laboratory Animals which greatly reduces the administrative burden for experimentation.

Zebrafish embryos have been used for acute toxicity testing (OECD, 2013), and for testing in the ToxCast™ (Padilla et al., 2012; Truong et al., 2014), and Tox21(Tice et al., 2013) high-throughput testing programs. In 2014, the National Toxicology Program (NTP) co-sponsored a Collaborative Workshop on Aquatic Models and 21st Century Toxicology, which highlighted the advantages and applications of zebrafish and other aquatic models in toxicity studies as well as impediments to their use (Hamm et al., 2019). One identified impediment, the lack of standardized protocols, led to the creation of NTP’s Systematic Evaluation of the Application of Zebrafish in Toxicology (SEAZIT, 2016) program. SEAZIT is a multipronged, multiyear initiative to support generation of fundamental knowledge for the use of zebrafish in toxicology research and provide the scientific basis for NTP’s future use of zebrafish in chemical toxicity screening. The initial phase of SEAZIT focused on cataloging common practices currently used in zebrafish assay protocols and involved interviews with zebrafish researchers in academic, federal, and industry laboratories (Planchart et al., 2016; Hamm et al., 2019). Interviews revealed a high degree of variability across design parameters, data collected, and analysis procedures (SEAZIT: Systematic Evaluation of the Application of Zebrafish in Toxicology). This variability was recognized as a barrier to research by preventing efficient integration of large quantities of data.

While zebrafish models are well-developed and accepted in toxicological studies, processes for generating and managing the resulting data have not kept pace and are a barrier to scientific advancement. Testing is performed across numerous laboratories, each of which has its own methodology and vocabulary for reporting results. These variations make it difficult, if not impossible, to compare and integrate data across laboratories. While these laboratories standardize their methodology internally, these standards are rarely applied across laboratories, resulting in different conventions for how the same phenotype is recorded. Differences in both granularity (e.g. “abnormal” caudal fin versus “curved” caudal fin) and notation (e.g., “caudal fin” vs “CAUD”) pose a problem for integrating and comparing data across laboratories and hinder corroboration of results from toxicological assessments. As a consequence, scientific progress is less efficient. Mismatches in granularity and vocabulary also hinder the generation of meta-analyses that require data integration. Workshops and publications following the establishment of SEAZIT explored the use of ontologies as a tool for overcoming these data integration barriers (Mattingly et al., 2016; Boyles et al., 2019; Thessen et al., 2020).

**FIGURE 1:**
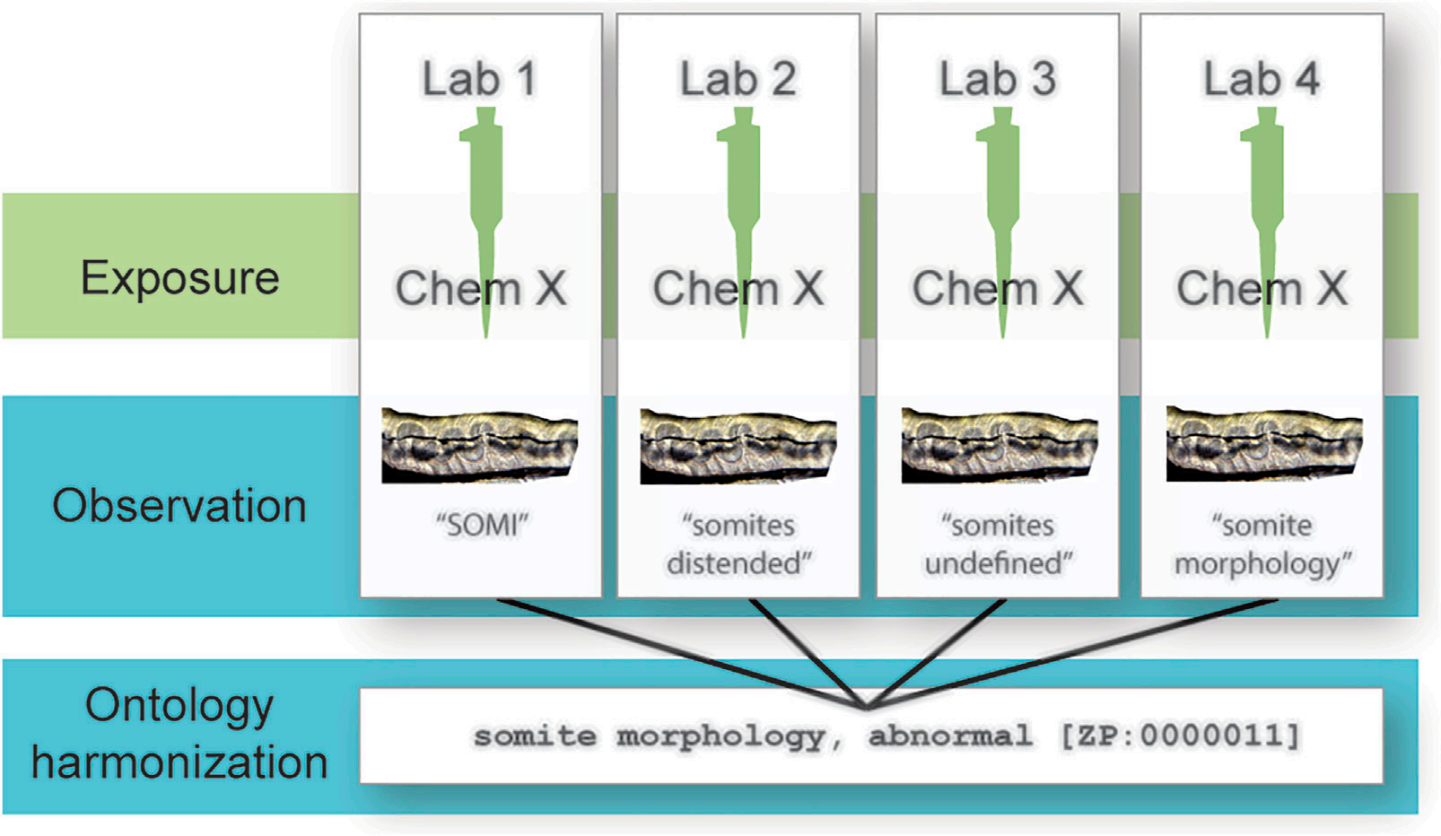
Reducing data heterogeneity with ontologies. Different laboratories test the same chemical and observe the same endpoint but report their observations differently according to each laboratory’s internal standard. Mapping these terms to an ontology reduces this heterogeneity and aids in data integration across laboratories.

An ontology is a formal, computational representation of knowledge in a particular domain. Terms are defined and arranged hierarchically. Relationships between terms are also defined, allowing logical inference and sophisticated queries. Ontologies are codified in a knowledge representation language, such as the Resource Description Framework (World Wide Web Consortium, 2014) or the Ontology Web Language (World Wide Web Consortium, 2012). Using ontology terms to report endpoints has the potential to reduce term heterogeneity and improve data integration across scale and granularity (Figure 1), thereby increasing the statistical power of toxicological assessments. The Zebrafish Phenotype Ontology (ZP) is a computational representation of knowledge about zebrafish phenotypes (Obophenotype, 2021) in the Zebrafish Information Network (ZFIN; Howe et al., 2021) and provides terms that can be used to record observed endpoints in toxicological studies. While the advantages of widespread adoption of an ontology such as ZP across laboratories seem clear, transitioning to a new standard presents practical challenges. These challenges can be addressed *via* a strategy to map laboratory conventions to an ontology, which would support automated integration of data without imposing a burden on laboratories. This strategy is employed by the Monarch Initiative, which integrates genotype and phenotype data across species for improved disease diagnosis (Shefchek et al., 2020).

To begin to address the issue of variability in reporting of zebrafish phenotype data, SEAZIT organized an April 2017 information session on “Implementation of Zebrafish Ontologies for Toxicological Screening” to review the state of the science for data analytics relevant to zebrafish screening studies (SEAZIT, 2017). Zebrafish researchers and data scientists discussed the current state of ontology usage in zebrafish toxicological screening, barriers to large-scale ontology use, and ways to encourage greater researcher adoption. Presentations by zebrafish researchers on the data collected in their laboratories emphasized the variability among researchers and the lack of consistent data annotation. However, discussions among the session participants revealed that, when examined from a data science perspective, many of these apparent differences could be parsed into the same or similar endpoints whose measurements differ only in time, methodology, or nomenclature. Based on that observation, participants discussed ways to collect endpoints along with relevant metadata to facilitate comparison among laboratories. Participants felt that a straightforward solution would be the creation of a web resource that would allow harmonized collection and representation of data, endpoints, and metadata - in other words, a zebrafish phenotype atlas, similar to the resource for the fish embryo acute toxicity test provided by von Hellfeld et al, (2020) and used in the Tanguay Lab (Tanguay Lab, 2021).

Here we discuss the use of biological ontologies as data integration infrastructure and present the results of a preliminary annotation exercise to investigate the impact of a hierarchical controlled vocabulary on the detection of toxicological endpoints in zebrafish larvae. To improve the current state of data reporting, we tested the hypothesis that providing ontology terms to raters would improve the consistency of endpoint reporting across laboratories in high-throughput zebrafish embryo-larval assays that assess larvae shortly after hatching. This manuscript describes the results of these experiments and discusses the computational advantages of annotating data with ontology terms.

## Methods

### Survey Participants

Survey participants, referred to as raters, were solicited *via* email from the authors’ professional networks. Every participant worked in a zebrafish laboratory with protocols for endpoint reporting. A total of 18 people from 14 government and academic research laboratories in multiple countries (Europe and North America) participated in at least one of the two surveys. Only the participants that took both surveys were included in the analysis.

### Surveys and Data Analysis

**FIGURE 2:**
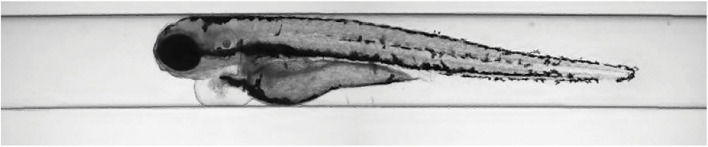
Example zebrafish larva image from the Vertebrate Automates Screening Technology System. Each survey participant was asked to annotate 24 of these images for each of two surveys.

Raters were asked to take two surveys. Survey 1 was a Google Form with 24 lateral images captured using the Vertebrate Automates Screening Technology System (Pardo-Martin et al., 2010), each image showing a single zebrafish at 96 h postfertilization (Figure 2). Raters were provided with a free-text response box and instructed to annotate each image with toxicological endpoints as they would in their laboratory. Survey 2 was a Google Spreadsheet with the same 24 images in a different order. Raters were provided with a multiple-choice list of 48 ontology terms and their definitions and were instructed to assign any terms that applied to each of the images.

The ontology terms provided in Survey 2 were selected from the endpoint annotations in Survey 1, with each rater-supplied endpoint annotation mapped to as specific an ontology term as possible from ZP using the ZP identifier represented as a CURIE (World Wide Web Consortium, 2010). All the mappings were performed by one person, referred to as the annotator, on the SEAZIT team. Each endpoint from Survey 1 was also categorized using a more general term. For example, a pericardial edema endpoint would be categorized as an abnormal heart endpoint. Survey results were analyzed using mean concordance and intraclass correlation to look for differences *1*) between the two surveys, *2*) between granular and general annotations, and *3*) to look at the differential effects of larvae and specific endpoints on annotation agreement. Concordance was assigned a binary value and measured agreement between a rater’s annotation and the majority. Concordance was grouped by rater and by annotation and mean concordance was computed for all data, Survey 1, and Survey 2. Intraclass correlation measured how much members of a group resemble each other. Here we treated larva, endpoint, and rater as random effects, then computed intraclass correlation of annotations for Survey 1 and Survey 2 using the rptR package (Stoffel et al., 2017). Both computations were done twice, once for the general and once for the granular annotations.

Both surveys provided to participants, the anonymized data collected, the statistical analysis, and the mapped ontology terms can be accessed at GitHub (https://github.com/diatomsRcool/zebrafish_phenotype_survey) (Thessen et al., 2021). After completion of the study, some participants mentioned difficulty in annotating images because the age of the larvae was unknown to them.

## Results

In Survey 1, raters used 1,748 unique terms to annotate 24 larvae (Tables 1, 2). These terms mapped to 48 traits from the Zebrafish Phenotype Ontology (21 general and 27 granular). We measured heterogeneity of description as the number of unique terms normalized to the number of times the trait was annotated (Table 2, Column 7: Terms per Tag), with a result of greater than 0.5 representing a high degree of heterogeneity and a result of less than 0.15 representing a high degree of homogeneity. Using this metric, abnormalities of the gut, pectoral fin, and otic vesicles were the most heterogeneously described (Table 2, highlighted in blue), while abnormalities of the trunk, snout, and heart were the most homogeneously described (Table 2, highlighted in green). These differences in heterogeneity could not be explained by differences in the number of granular traits for each general trait (Table 2, Column 5: Child Traits), the number of times the general trait was used (Table 2, Column 3: Total Tags), or the number of larvae displaying that trait (Table 2, Column 6: Number of Larvae). Not only did raters disagree on the terms used to describe endpoints, but also differed on which endpoints were worthy of note and their general characterization. For example, there were larvae where only some raters reported a heart abnormality as an endpoint. Raters also disagreed in their identification of whether an image depicted a normal or a dead larva.

**TABLE 1:** Data for example trait: abnormal tail.

General term	Granular term	CURIE	Verbatim annotation by participant
abnormal tail	—	ZP:0001129	Malformation tail
abnormal tail	—	ZP:0001129	tail deformation
abnormal tail	—	ZP:0001129	malformation tail
abnormal tail	—	ZP:0001129	length of the tail
abnormal tail	abnormal tail fin	ZP:0004969	caudal fin malformation
abnormal tail	abnormal tail fin	ZP:0004969	caudal fin malformations
abnormal tail	abnormal tail fin	ZP:0004969	malformed caudal fin
abnormal tail	abnormal tail fin	ZP:0004969	Cfin
abnormal tail	abnormal tail fin	ZP:0004969	cfin
abnormal tail	abnormal tail fin	ZP:0004969	Malformation tail fin
abnormal tail	abnormal tail fin	ZP:0004969	Tail End Vacuolization
abnormal tail	abnormal tail fin	ZP:0004969	ruffled fin
abnormal tail	abnormal tail fin	ZP:0004969	tail tip necrosis
abnormal tail	abnormal tail fin	ZP:0004969	vacuolization in end of tail
abnormal tail	abnormally curved tail	ZP:0010319	Curved tail
abnormal tail	abnormally curved tail	ZP:0010319	Slightly Curved Tail
abnormal tail	abnormally curved tail	ZP:0010319	bent tail tip
abnormal tail	abnormally curved tail	ZP:0010319	C tail
abnormal tail	abnormally curved tail	ZP:0010319	C-tail
abnormal tail	abnormally curved tail	ZP:0010319	bend tail
abnormal tail	abnormally curved tail	ZP:0010319	curved tail
abnormal tail	abnormally curved tail	ZP:0010319	curved tail tip
abnormal tail	abnormally curved tail	ZP:0010319	slightly curved tail
abnormal tail	abnormally curved tail	ZP:0010319	slight tail curve
abnormal tail	abnormally curved tail	ZP:0010319	tail curve
abnormal tail	abnormally curved tail	ZP:0010319	tail tip curve
abnormal tail	abnormally curved tail	ZP:0010319	tail bending
abnormal tail	abnormally curved tail	ZP:0010319	abnormal tail curvature
abnormal tail	abnormally short tail	ZP:0001130	Possible Short Tail
abnormal tail	abnormally short tail	ZP:0001130	short tail
abnormal tail	abnormally short tail	ZP:0001130	possible short tail
abnormal tail	abnormally short tail	ZP:0001130	reduced tail length

**TABLE 2:** Survey 1: analytical summary.

General trait	CURIE	Total tags^a^	Unique terms^b^	Granular child traits^c^	Number of larva^d^	Terms per tag^e^	Terms per trait^f^
abnormal	ZP:0005632	24	7	0	22	0.29	7
abnormal axis	ZP:0127724	71	15	1	12	0.21	8
abnormal body length	ZP:0012799	82	16	1	18	0.20	8
abnormal brain	ZP:0000100	40	7	0	16	0.18	7
abnormal eye	ZP:0000943	77	12	1	15	0.16	6
abnormal gut	ZP:0002008	8	6	1	4	0.75	3
abnormal head	ZP:0001609	127	30	3	23	0.24	8
abnormal heart	ZP:0000107	249	28	2	20	0.11	9
abnormal jaw	ZP:0007203	153	24	2	24	0.16	8
abnormal notochord	ZP:0000624	49	22	4	8	0.45	4
abnormal otic vesicle	ZP:0001601	41	21	0	8	0.51	21
abnormal pectoral fin	ZP:0001610	14	12	0	13	0.86	12
abnormal pigmentation	ZP:0015121	29	12	1	12	0.41	6
abnormal snout	ZP:0014550	78	5	0	22	0.06	5
abnormal swim bladder	ZP:0127709	221	34	3	20	0.15	9
abnormal tail	ZP:0001129	79	33	3	16	0.42	8
abnormal trunk	ZP:0003437	43	3	0	12	0.07	3
abnormal yolk	ZP:0002676	274	53	5	23	0.19	9
dead	ZP:0000306	2	2	0	1	1.00	2
necrosis	ZP:0000398	12	8	0	5	0.67	8
normal		51	17	0	9	0.33	17
hatched		24	1			0.04	

Annotations with green color are those with a high degree of homogeneity and annotations with a blue color are ones that had a high degree of heterogeneity.

a The number of times the trait, using a general or granular term, was tagged across all larvae and annotators.

b The number of unique strings used to describe the trait across all larvae and annotators.

c The number of granular traits that fall under each general trait.

d The number of larvae to which the trait was applied at least once.

e The number of unique terms normalized to the number of times the trait was annotated.

f The number of unique terms normalized to the number of general and granular traits.

For the general terms, the rater mean concordance went up from 86.0% +/− 4.1% in Survey 1 to 86.3% +/− 2.5% in Survey 2. For granular terms, the rater mean concordance went down from 92.1% +/− 2.6 to 90.3% +/− 3.1%. Even though we used the results from Survey 1 to select terms provided for Survey 2, five raters reported that they were not provided all the terms that they needed to fully annotate 19 of the 24 larvae.

**FIGURE 3:**
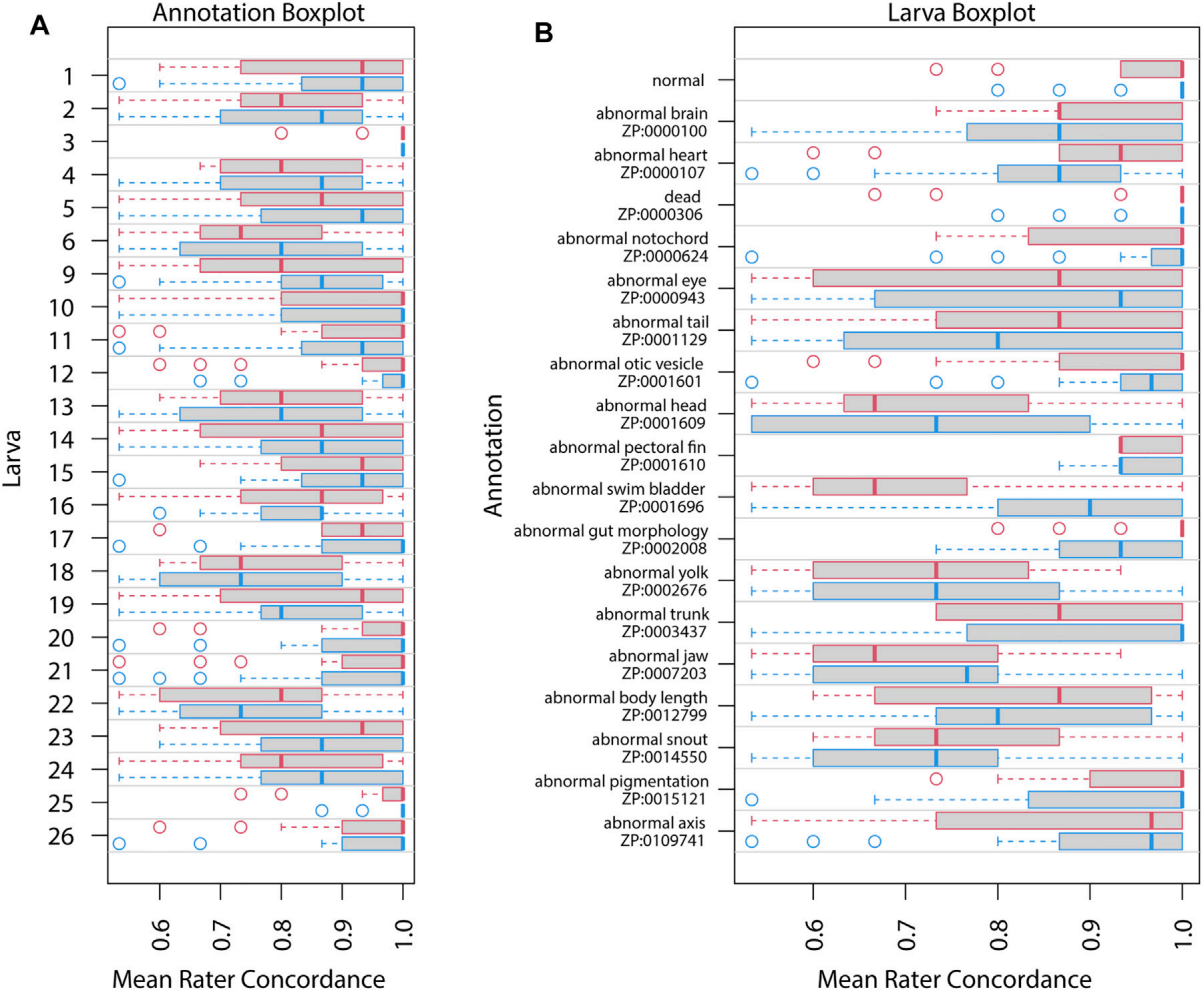
Mean rater concordance using general phenotype terms. These boxplots show the mean concordance (*x* axis and red or blue bar in shaded box) of the raters by larva **(A)** or by annotation **(B)** with interquartile range indicated by shaded area (first to third quantiles). Data from Survey 1 are in red and from Survey 2 are in blue. The dashed whiskers denote the data that are within 1.5 times the interquartile range, with circles annotating data outside that range. Please note that larvae 7 and 8 did not exist. No data were discarded.

**FIGURE 4:**
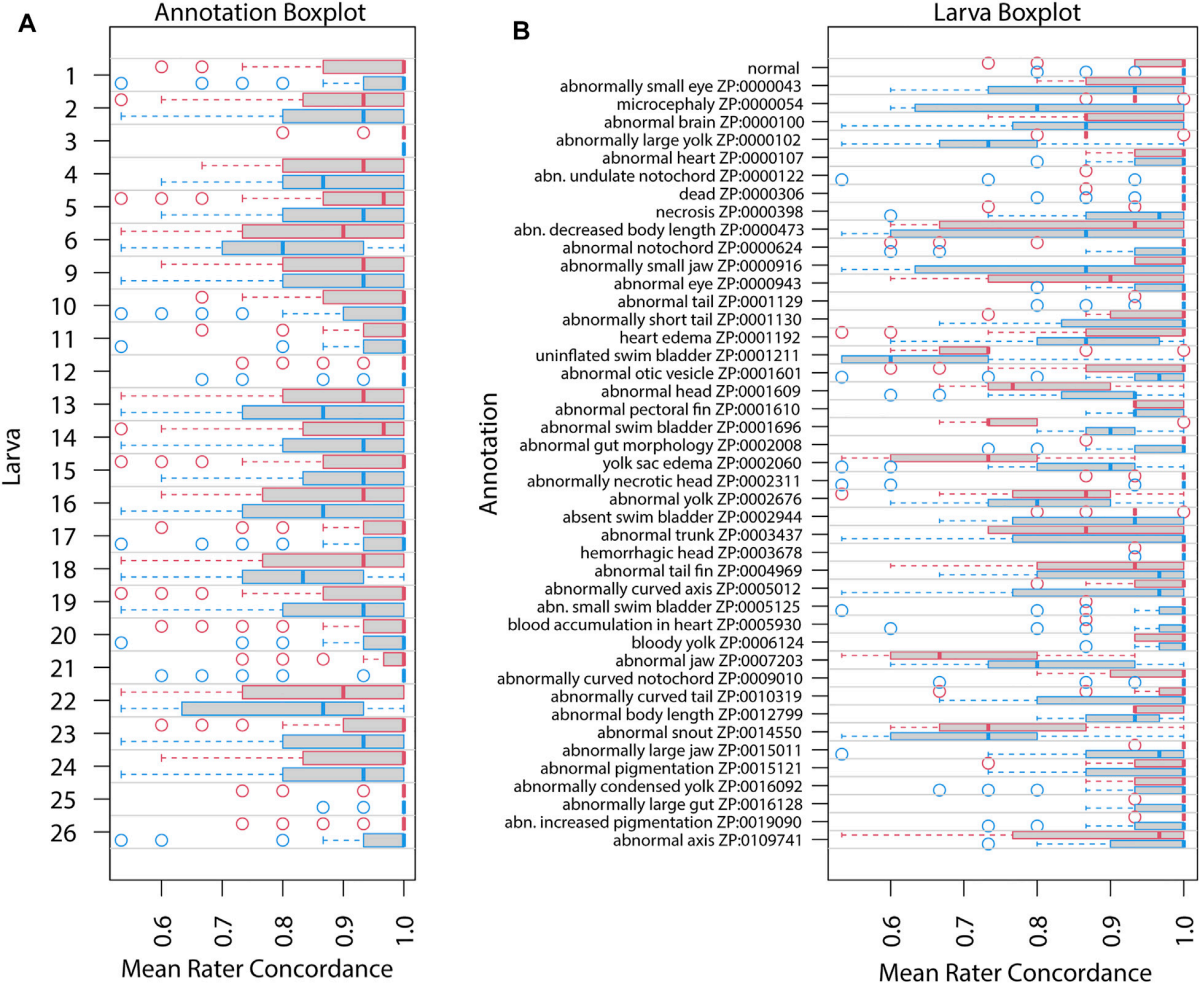
Mean rater concordance using granular phenotype terms. These boxplots show the mean concordance (*x* axis and red or blue bar in shaded box) of the raters by larva **(A)** or by annotation **(B)** with interquartile range indicated by shaded area (first to third quantiles). Data from Survey 1 are in red and from Survey 2 are in blue. The dashed whiskers denote the data that are within 1.5 times the interquartile range, with circles annotating data outside that range. Please note that larvae 7 and 8 did not exist. No data were discarded.

Repeatability as estimated by intraclass correlation (ICC) increased in Survey 2 (Table 3) for all except the granular annotation. The estimated ICC for general annotation increased, likely reflecting uneven difficulty across annotation (Figures 3, 4). However, all of these ICC values are low and indicate that very little variance is explained by these groupings.

**TABLE 3:** Intraclass correlation repeatability estimate.

Annotation granularity	Grouping factor	Survey	
		1	2
General	Larva	0.092	0.159
	Annotation	0.208	0.263
	Rater	0.043	0.047
Granular	Larva	0.038	0.068
	Annotation	0.150	0.111
	Rater	0.016	0.019

Figures 3, 4 show distributions of concordance among raters, using general and granular phenotype terms, respectively, from both Survey 1 and Survey 2. These data show differences across both larva (Figures 3A, 4A) and annotation (ZP CURIE; Figures 3B, 4B), as exhibited by shifts in both mean concordance and interquartile range (i.e., across the horizontal axes). For example, certain larvae (3, 12, 25, 26) were consistently easier to annotate across both surveys (Figures 3A, 4A). This result is related to the observation that certain endpoints were more consistently reported (i.e., higher observed concordance) in Survey 2 (Figures 3B, 4B). Raters found it easier to identify normal larva, abnormal swim bladders, abnormal notochords, abnormal somites, abnormal eyes, abnormal heads, abnormal jaws, abnormal axes, and abnormal trunks as well as yolk sac edema. The raters found most of the endpoints represented by granular terms more difficult to consistently annotate in Survey 2, the exceptions being the endpoints “yolk sac edema” and “abnormal tail fin” (Figure 4B).

**FIGURE 5:**
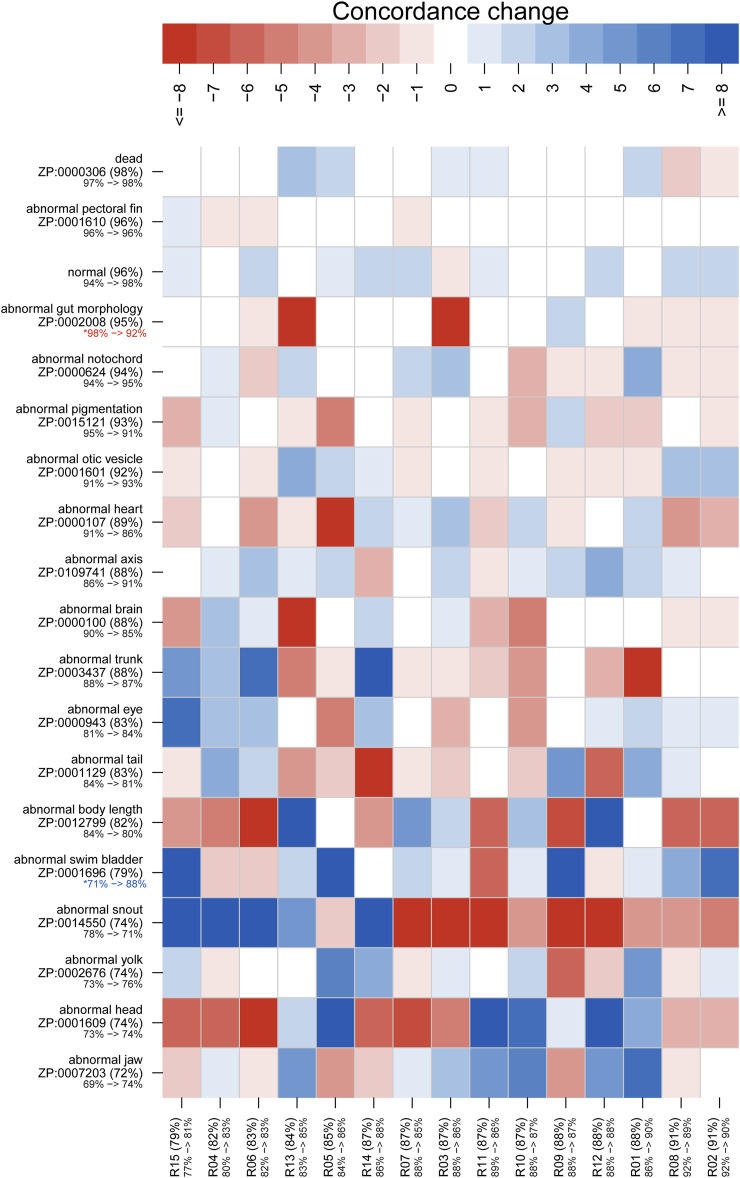
Concordance change for general phenotype terms. Concordance here represents the frequency for which a particular rater (identified along *x* axis) made the same annotation as the majority of raters. The “concordance change” is calculated as the number of concordant annotations for Survey 1 subtracted from those for Survey 2 (maximum range is from −24 to 24). An increase in concordance is indicated by blue and a decrease is indicated by red. Both the annotation and rater labels have the overall mean concordance for both surveys in parentheses, with a color-coded change in mean concordance from Survey 1 to Survey 2 below. Note that the lower bound for the annotation mean concordance is 50%, but the rater lower bound is 0%. Axes are sorted by overall mean concordance values. Significant changes in concordance as determined by Fisher's exact tests are indicated by an asterisk.

**FIGURE 6:**
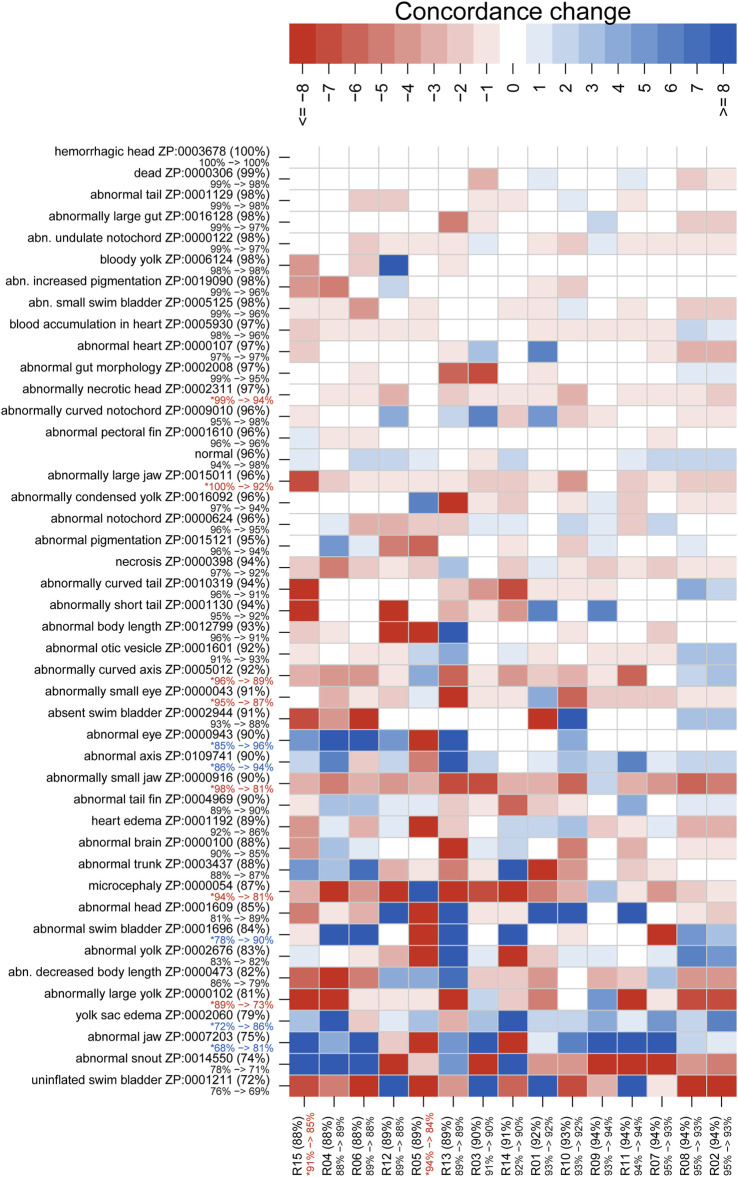
Concordance change for granular phenotype terms. Concordance here means the rater made the same annotation as the majority of raters. The “concordance change” is the difference between the number of concordant calls for Survey 2 and those for Survey 1 (maximum values would range from −24 to 24). An increase in concordance is indicated by blue and a decrease is indicated by red. The annotation and rater labels have the overall mean concordance in parentheses (combines both surveys), and a color-coded change in mean concordance from Survey 1 to Survey 2 just below. Note that the lower bound for the annotation mean concordance is 50%, but the rater lower bound is 0%. Axes are sorted by overall mean concordance values. Significant changes in concordance as determined by Fisher's exact tests are indicated by an asterisk.

Figures 5, 6 illustrate concordance change for general and granular phenotype terms, respectively, represented as the frequency for which a particular rater made the same annotation as the majority of raters. The change in concordance was computed as the difference in mean concordance (*see* Methods) between surveys. The change in concordance of general phenotype terms from Survey 1 to Survey 2 (Figure 5) was statistically significant for abnormal swim bladder (+17), and abnormal gut morphology (−6%). Changes in individual rater concordance varied from +4% to −3%, but was not statistically significant. Some terms, like abnormal snout (ZP:0014550) exhibited a wide variation in concordance among raters. The change in concordance of granular phenotype terms from Survey 1 to Survey 2 (Figure 6) was statistically significant for abnormally necrotic head (−5%), abnormal jaw (+13%), yolk sac edema (+14%), abnormally large yolk (−16%), abnormal swim bladder (+12%), abnormally small head (−13%), abnormally small jaw (−17%), abnormal axis (+8%), abnormally small eye (−8%), abnormally curved axis (−7%), abnormally large jaw (−8%), and abnormal eye (+11%). Changes in individual rater concordance varied from +1% to −10%, but were only statistically significantly decreased for two.

**FIGURE 7:**
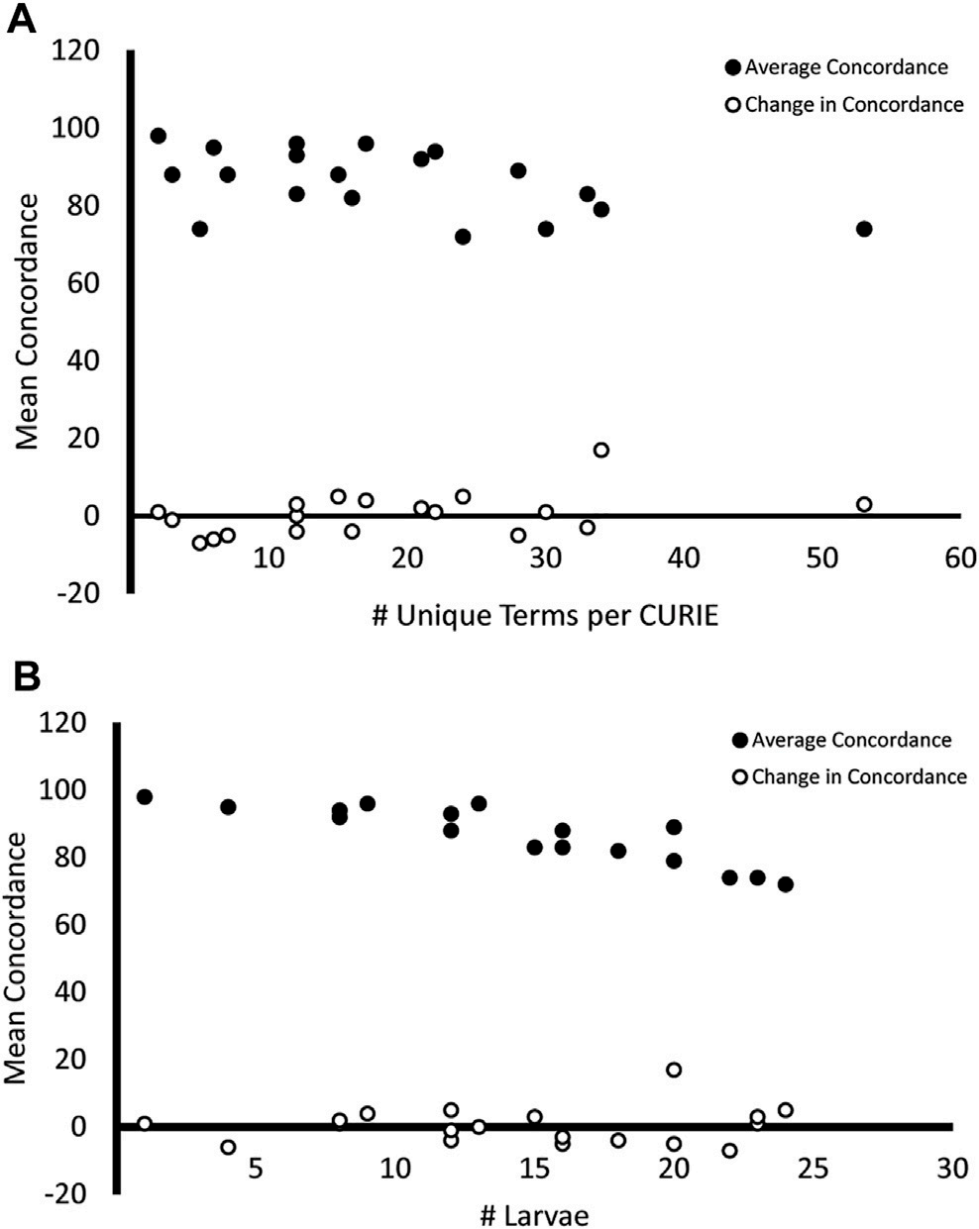
Mean concordance and variability in endpoint reporting. Endpoints that were described using a higher number of unique terms **(A)** and were observed in more larvae **(B)** had a lower mean concordance across both surveys (filled circles). The change in concordance from Survey 1 to Survey 2 did not share this relationship (open circles).

Figure 7 shows that there are negative correlations between the mean concordance for each general term and both the number of unique terms used to describe each trait (Figure 7A, Pearson Correlation Coefficient = −0.51) and the number of larvae displaying each trait (Figure 7B, Pearson Correlation Coefficient = −0.89). This, in combination with the correlation between the number of unique terms and mean concordance (Figure 7A, Pearson Correlation Coefficient = 0.42) and the number of larvae and mean concordance (Figure 7B, Pearson Correlation Coefficient = 0.12) suggests that the variability we observed in endpoint reporting was driven more by the reporting methodology than by the endpoint.

## Discussion

In this project, the requirement in Survey 2 that raters use ontology terms from ZP resulted in greater consistency in some categories of endpoint reporting across laboratories compared to the free-text responses obtained from Survey 1 by reducing the heterogeneity of terms. However, we still observed substantial variation in concordance across larvae, raters, and annotations in responses to both surveys.

The most notable result of the study was the overall decrease in repeatability and mean concordance in the granular endpoint annotations from Survey 1 to Survey 2. This is likely the result of raters and their labs using imprecise terms to describe endpoints in Survey 1. The mapping of free-text endpoints to ontology terms was done by one annotator without consulting the participant (to avoid affecting their answers in Survey 2). As a result, the ontology term reflects the annotator’s interpretation of the meaning of the text used, which may or may not have captured the participant’s true intent. It is important to note that there was no training provided to participants in the current study. It is reasonable to assume that training would have improved the participants understanding of the new ontology terms and likely further improved repeatability and mean concordance.

A brief survey of endpoint reporting terms used by four laboratories showed that ambiguous usage and overloading of terms are common. For example, a participant might conflate yolk sac enlargement and yolk sac edema into a single “YSE” endpoint, although they are two distinct ontology terms with precise definitions. In this study, “YSE” was mapped to “yolk sac edema” and that term was provided in Survey 2. Thus, when presented with a list of precise ontology terms in Survey 2, the same participant might choose “yolk sac edema” (granular) or simply “abnormal yolk” (general) or report that they do not see their desired term. Indeed, five raters reported this to be the case. This mismatch of meaning would have a greater effect on the reporting consistency of granular terms than on the general terms and could explain these results.

The variation in concordance between Surveys 1 and 2 (Figures 5, 6) demonstrate that use of a standard would not improve consistency in reporting for all endpoints and not all raters focused on the same features. For example, abnormalities of the swim bladder, which showed substantial changes in concordance from Survey 1 to Survey 2 and had highly variable concordance across raters, represents a type of endpoint that has a high potential for improved consistency in reporting across labs when a well-developed standard is applied. These types of endpoints were described in Survey 1 using ambiguous terms (Table 2; except for abnormal snout) and showed a pattern of decreased concordance for terms that appeared in more larva. Conversely, endpoints like abnormal pectoral fin or hemorrhagic head showed little to no change in concordance between surveys, suggesting that both the terms used and the features observed are already clear and stable. The overall negative correlation (Figure 7) between the heterogeneity of terms used to describe an endpoint and both the number of times a particular endpoint appears, and the mean concordance of that endpoint argues that methodology is a significant source of variability in endpoint reporting. Thus, a broad effort to standardize endpoint reporting and annotator training is needed.

These data suggest that providing ontology terms to raters will improve agreement of feature annotation among researchers, but a larger data set is needed to make definitive statements about inter-rater reliability for qualitative items (Kappa interpretation) by systematically characterizing effects of prevalence across a wider endpoint and larvae set (Sim and Wright, 2005). Overall, these data show a trend toward improvement in consistency and interoperability and justify continued investigation with more participants.

**FIGURE 8:**
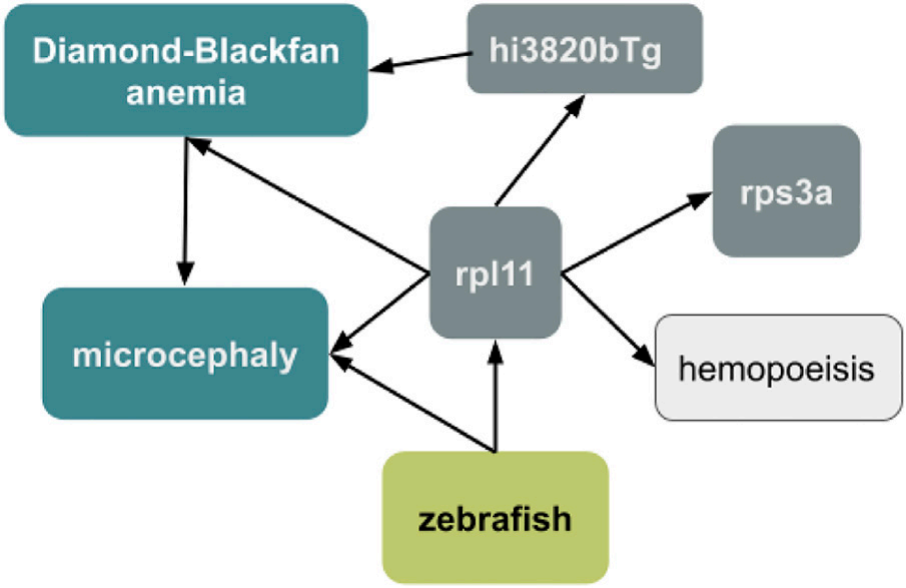
Expanding a data set using a knowledge graph. The zebrafish endpoint “microcephaly” can be used to query the Monarch knowledge graph to find relevant genes (rpl11 and rps3a), variants (hi3820bTg), diseases (Diamond-Blackfan anemia), and biological processes (hemopoeisis) to enrich the data set and generate new hypotheses.

When combined with a semantic model, the use of ontology terms can facilitate the incorporation of data into knowledge graphs (Figure 8). A semantic model is a data model that includes defined, formal logic relationships between entities. The semantic model we are testing for representing exposures was developed in collaboration with the larger exposure science and toxicology community, including representatives from the AOP ontology and the US EPA. This permits the synthesis of endpoints as constellations of related biological outcomes. For example, querying a biomedical knowledge graph like Monarch for the microcephaly phenotype in zebrafish (ZP:0000054) identifies relevant genes, variants, diseases, and biological processes. An ontology-based synthesis would enable different types of analyses such as phenotype similarity comparisons with data in the Monarch Database to identify new gene candidates that are related to the response. Unfortunately, the use of imprecise terms can restrict annotations to more general terms, such as abnormal head instead of microcephaly. It is unclear if a knowledge graph query using a more general term would yield equally useful results.

The field of translational medicine uses data from basic science approaches, such as high-throughput screening, to generate insights that advance health care (Kaufman and Curl, 2019). This activity relies on data infrastructure such as knowledge graphs to standardize and integrate similar data and link related data across studies and disciplines (Nicholson and Greene, 2020). Data about toxicological endpoints can play an important role in translational science for public health if standards for these data are developed and adopted (Canzler et al., 2020). Toward this goal, community efforts have identified four gaps that prevent the adoption and use of methods that would facilitate the creation computable toxicological data (Thessen et al., 2020). Two of those four gaps, lack of ontology terms and lack of a semantic model, are relevant to the work presented in this study. This study suggests not only that use of standards can improve immediate data quality, but their use can also contribute to unanticipated insights *via* translational science.

Future work will include developing protocols for high-throughput endpoint reporting that are equipped for mapping to specific ontology terms. Developing such protocols will be a challenge due to the need of high-throughput screening to strike a balance between precision and speed. We are engaged in fostering an environmental health sciences community to drive creation of a semantic infrastructure for environmental exposure data. Activities of this community include bottom-up development of use cases and competency questions to support definition of standards.

Based on the results reported here, we also recommend the development of a zebrafish phenotype atlas that would include a standard set of well-described and documented endpoints for use in high-throughput screening that would accommodate the needs of diverse laboratories. A recently developed catalog of morphological zebrafish endpoints for the fish embryo toxicity test is an excellent resource (Mattingly et al., 2016). We intend to build on this work to map ZP CURIEs and individual laboratory phenotype definitions to this standard with a focus on the endpoints that have been identified here as having heterogeneous reporting. If we are successful in these two efforts, high-throughput screening-derived toxicological data sets will become more valuable by their increased replication and integration with genomic and phenomic data sets. Finally, having this new information available in an appropriately interconnected database will allow comparisons between model organisms and human clinical data, as is available *via* the Monarch Initiative (Shefchek et al., 2020).

## Data Availability

The datasets presented in this study can be found in an online repository. The name of the repository can be found below: https://github.com/diatomsRcool/zebrafish_phenotype_survey.
